# The Orbicularis Oris, and the Muscles of Expression

**Published:** 1897-01

**Authors:** W. C. Barrett

**Affiliations:** Buffalo, N. Y.


### THE DENTAL REGISTER.

VOL. LI.] JANUARY, 1897. [No. 1.

### COMMUNICATIONS.

### The Orbicularis Oris, and the Muscles of Expression.

BY W. C. BARRETT, M.D., D.D.S., BUFFALO, N. Y.

Read before the American Dental Association, August, 1896.

There are a thousand millions of people living upon the earth, and no two of them resemble each other so closely that they can not be distinguished. The characteristic differences are mainly found in the facial features, and in them the diversities are produced, partially by modifications of the facial and cranial bones, but more especially by variations in the development of the facial muscles, the most expressive of which is the orbicularis oris.

It follows, then, that our principal means for the identification of our friends and acquaintances is in the special development and functional action of this, the most wonderful of all the muscles of the body. When we consider the marvelous diversity of the expressions which the human countenance can assume, when we remember that pleasure, grief, rage, scorn, love, indifference, pride, jealousy, devotion, elation, melancholy, fear, hate, hope, and a hundred other sentiments and passions, with all their modifications, can be clearly and unmistakably expressed by mere contractions and relaxations of these muscles, we begin to comprehend something of the amazing variety of the combinations that may be formed by their union. The more compound a set of muscles, the greater the range of its mobility, usually, however, at the expense of its muscular power. Even the features of the stolid, unimpressionable, phlegmatic boor clearly present, at times of excitement, the workings of his uneducated, untrained mind. When we compare with this the language in which the muscles of the trained pantomimist speak, we get a glimpse of their capabilities.

Nor is it alone upon the voluntary muscular contractions that the actor relies for the portrayal of human emotions. He uses appliances for the mechanical distortion of them, that he may the more readily assume special expressions. It is well known that the tragedian and the comedian introduce material into the mouth for the distention of certain muscles, holding them in permanent distortion in their "make up," that by the action of certain other muscles in combination with them, exaggerated expressions may be produced. An invisible hair or thread is used to draw up or down certain facial attachments, thus to produce some special combination. Men so disguise themselves by artificial means that they are not readily recognized by their friends. Criminals, by certain very simple surgical operations, so alter their whole expression that detectives are unable to identify them. Squint, or cross-eye, can be produced or obliterated almost at will. Slight permanent contractions of certain muscles can be induced by simple operative procedures that shall entirely change the expression of the whole face. We, as dentists, know the marvelous modifications that can be brought about through a change in the position of the teeth. We have witnessed the complete mutilation that has been caused by the insertion of an unskillfully-constructed artificial denture. I could not, then, select a subject that should more directly appeal to those who are constantly engaged in working upon the human face, than to present before them a brief study of one of these expressive organs.

The orbicularis oris is the most complicated and involved of the muscles of the human body, whether we consider it from its anatomical or functional aspect. It is commonly considered a sphincter, but it is very far removed from this class in that none of its fibers are continuous about the cavity which it surrounds. It is not a simple muscle, but it is made up by the intermingling and intercommunicating of a large number of facial muscles. In fact, the distinctive fibers of the orbicularis are comparatively few, passing only across the lips, and not around them. The compression of the mouth is not even produced as in the eye, in which the orbicularis palpebrarum passes nearly around. The mouth can not be completely pursed up as with a true sphincter, but it can be drawn together by the combined action of a considerable number of muscles. Its expressiveness is not produced by a simple muscular action, nor can any of the passions be portrayed by any one set of fibers. The marvelous changes are brought about by combinations made possible through the intercommunications already referred to.

The orbicularis oris is the common meeting-ground of all the muscles of the facial expression. Fibers from each and every one of them communicate, either primarily or secondarily, with the true orbicular fibers. Even the muscular portion of such a distant one as the occipito-frontalis has its means of communication, and from the clavicle to the coronal suture there is a functional union of all the expressive muscles of the face and head. The mingling of the fibers of all these muscles makes the structure of the orbicularis very complex. Muscles like the depressors and the levators, the filaments of which are arranged at right angles to the margins of the lips, crossing it, give vertical fibers. Those of the buccinator, risorius, etc., added to the longitudinal fibers of the orbicularis proper, furnish transverse filaments, while a third set runs diagonally across, obliquely between the other bundles, from before backward and from the skin to the internal mucous membrane.

The muscle, as a whole, may be divided into the internal or labial, and the external or facial portions, according to whether the layers are mainly composed of the fibers of the superficial or deep muscles, but no accurate line of demarcation can be drawn, because of the diagonal fibers that interlace. The deep portion has but a restricted attachment to the bone beneath, that of the upper lip having the naso-labial slips, which give the prominence that forms the two vertical ridges near its center, dropping down from the cartilage of the nose, and another in the incisive fossa, while that of the lower lip is merely attached at the incisive fossa on each side. The superficial part of the muscle has no bony attachment whatever, but is held in position by the diverging muscles of which it is composed. This gives extreme mobility to the whole mouth, the lack of bony attachment accentuating it, for, if the superficial fibers are strongly contracted and the diagonal ones relaxed, the lips are protruded, while if the deeper layers and the oblique ones are contracted the lips are drawn against the teeth.

The buccinator belongs to the deeper layer, and its fibers decussating or crossing at the angles of the mouth, make up a large part of the transverse portion of the orbicularis. It has its origin along the distal line of the alveolar process of both the upper and the lower jaws, and in the pterygo-maxillary ligament behind, many of the fibers from the upper part crossing and forming a part of the orbicularis below, while those of the inferior part cross to be inserted in the upper lip. These decussating fibers are attached to the alveolus as far forward as the margin of the canine fossa, and they should dominate the shape of any artificial denture at this point.

The Levator anguli oris, the Levator menti and the quadratus menti belong to the intermediate layer, while the Levator labii proprius, the zygomaticus major and minor, the triangularis menti, the risorius and the Levator labii superioris alæque nasi, with the fibers of the platysma myoides, make up the superficial layer. But certain fibers of all these muscles cross and, accompanied by portions of the deep fascia, penetrate even to bony attachments.

It is not, however, the anatomy of these muscles that so much interests us as their function, and I desire to indicate in what way the expression of the countenance may be effected by malpositions of the teeth, or by unskillful operations on the part of the dentist.

First, let us consider the lavator labii superioris alæque nasi. The action of this muscle is to raise and slightly evert the inner half of the lip, and at the same time to lift the wing of the nose, and it produces an expression of disgust. It is especially used in sneering. In certain instances in which the central incisors are especially prominent while the laterals are retracted, we have that peculiarly-impudent and deriding aspect that is sometimes called the "squirrel," or "rat face." If an artificial plate be too prominent beneath it, there may be produced the vacant, idiotic look, induced by the compression of the oblique fibers of the orbicularis oris, and the consequent eversion of the labial edge.

A little nearer the angle of the lips and above the canine teeth, but upon the superficial labial surface, lies the levator labii proprius. The action of this muscle is quite complex. With the levator labii superioris alæque nasi it raises the lip, everts it somewhat, and gives a peculiar sneering expression to the countenance. The carnivoræ, in snarling, bring this muscle into play, and thus expose the canine teeth. In connection with the depressor anguli oris, or triangularis menti, and the orbicularis palpebrarum, it is especially used in crying. The closure of the eye by the contraction of the latter muscle assists in raising the lip, while the triangularis menti draws the mouth into the shape of a parallelogram, and gives a peculiarly lugubrious expression to the face. If the cuspids be unduly prominent, or if a plate lifts the muscle too much, the consequence may be, when the other muscles are at rest, a permanent sneer. With the contraction of the orbicularis palpebrarum and the triangularis menti, the face has an expression of extreme sadness.

The buccinator, as a muscle of expression, acts secondarily. From the peculiar direction of its fibers, when it is in contraction, the muscles of the deep layer are pursed up at the corners of the mouth, while those of the more superficial layer are peculiarly puckered by the consequent drawing of the levator and depressor anguli oris. A deep wrinkle is seen at the angle, while the risorius is placed upon a stretch, and an insincere and mocking expression is the result. This will be induced when an artificial denture is inserted that has no depression at the canine fossa. The superior decussating fibers of the buccinator are placed upon a stretch, and a peculiarly-disagreeable expression thereby induced. The same abnormality of the natural denture, or the same improperly-constructed artificial plate that thus unduly distends the buccinator, will act upon the levator anguli oris and intensify the disfigurement. This muscle raises the corner of the mouth, and at the same time draws it inward. When too much distended there must be a continued muscular effort on the part of the triangularis menti to keep the corners of the lips in place, and this results in a painfully-strained appearance and adds to the distortion.

The zygomaticus, major and minor, are the muscles most used in smiling, as they draw the angle of the mouth upward and outward. When the zygomaticus major is strongly contracted, it draws together the tissue about the point of its origin and causes a fullness in front of the malar bone. If now the orbicularis palpebrarum be contracted, the tissue between the origin of the zygomaticus and the outer canthus of the eye is peculiarly wrinkled, producing what are called "crow's feet," a disfigurement which ladies especially most earnestly desire to avoid. If both the zygomatics are placed upon a stretch by undue distention, the mouth is placed upon a broad grin. If, on the other hand, they are unduly relaxed, the triangularis menti draws down the corners of the mouth, and gives the opposite expression.

The risorius has its origin in the masseter, and its action is to draw the corners of the mouth directly outward. It gets its name from the former supposition that it was the muscle of laughter, but all late anatomists know that this was a mistake. When it is contracted it gives an expression of pain. This is the case in tetanus, and hence the appearance in that condition is called the risus sardonicus. If the first molar in either the natural or an artificial denture be placed either within or without the proper position, the *risorius* may be caused to give a very peculiar and painful expression.

The platysma myoides is not as much affected by the dentition as some of the other muscles. Yet there are few whose action is more complex. Arising as it does from the deep facia of the upper part of the chest and neck, it is involved in all of the muscular movements of the important pectoralis major, the deltoid and sterno-cleido mastoid. Its long bundles of fibers continue to the border of the jaw, where some of them are inserted. But others cross the lower jaw, and continuing beneath the triangularis and the quadratus menti are lost in the orbicularis oris, while the fibers crossing the angles of the mouth are mingled with and lost in the fibers of the levator labii proprius and the levator labii superioris alæque nasi. Hence, any unusnal exertion of the pectoralis and the deltoid exhibits itself in the face. In heavy lifting, the corners of the lips are drawn down, and even the wing of the nose depressed by the strain placed upon the platysma, through its origin in the facia of the muscles of the arm and chest, and its connection with the orbicularis oris and the levator alæ nasi. It can not then be lost sight of in arranging the teeth, and its action upon the orbicularis oris needs careful study by the artistic dentist.

The depressor anguli oris is, of course, affected by anything that unduly contracts the levator anguli oris and the zygomatics. If, on the other hand, these muscles are left too lax, the depressor or the triangularis menti draws the mouth out of position. If the lower teeth are too prominent, or if an inferior artificial denture places these muscles upon a stretch, it may be easily seen that not only will the symmetry and proper expression of the lower lip be destroyed, but that the opposing muscles of the upper lip will draw that also out of place. Especially must the natural depression of the canine fossa be retained, that the *triangularis menti* may not be distorted, and so the natural expression be entirely lost.

A very disagreeable fullness of the lower lip may be induced if an artificial plate too much distends the quadratus menti or the depressor labii inferioris, and the lavator menti or lavator labii inferioris. If the latter, especially, be strained by undue fullness near the center of the lower lip, it induces a peculiarly haughty, supercilious expression, which may do the wearer of it great injustice. This appearance may be brought about by mal-position of the teeth, irrespective of either the natural or the artificial alveolus. If, for instance, the lower edge of an inferior plate be too thick at the incisive fossa, the origin of the levator menti, the point of the chin will be disagreeably drawn up. If now the teeth be placed too far back, the natural fullness of the orbicularis is not preserved, and the decussating fibers of the buccinator and those of the platysma will draw in the margin of the lip, and a peculiarly-dogged, obstinate expression will be the consequence.

If a plate be too full at the canine fossa of the upper jaw, the levator anguli oris is distended and the corner of the mouth is raised, while at the same time the descending fibers of the buccinator draw up the inferior angle of the mouth, the risorius is pulled out of position, and a half-sneering, half-weeping expression is seen. At the same time the zygomatics may be relaxed by the lifting of the lip, and there will be a distortion of the tissues covering the malar bone. Hence, by an artificial denture that has an undue prominence at one point, every expression of the face may be changed, and not a muscle that is connected with the orbicularis oris remain at rest in its natural state.

It would be interesting at this point to take up the comparative muscular anatomy of different orders of animals, and determine the character of each as expressed in the face. There is a peculiar development of the muscles about the mouth of each one, and it is always indicative of their individual or class-traits. In the carnivora, for instance, the levators of the upper lip are largely developed. This gives them the peculiarly savage expression that is so threatening when the anger of the animal is aroused.

In the rodentia the prominence of the central incisors brings the levators proprius into prominence, and gives them the impudent, saucy expression that is characteristic of that order. In the leporidæ, or hares, this is modified by partial separation of the superior labial muscles, the so-called "hare-lip."

In the ungulatæ the absence of developed canines in many of the families of this order is partially compensated for by the peculiar arrangement of the six incisors, while the corners of the mouth are so far back that the angular muscles are partially inactive. Yet the arrangement is plainly observable in the characteristic expression, this order being, except in the case of certain sub-orders, timid and fearful. The presence or absence of the cuspid changes the whole expression, and the horse, which has cuspid teeth, may, by close and experienced observers, be distinguished from the mare, which has none. But this study, although intensely interesting, is foreign to the objects of this paper, and I will not enlarge upon it. I could not, however, resist the impulse to advert to it.

I should be glad also to take up at greater length the question of functional anatomy, but time forbids. I can but superficially present my subject at best, as much time should be spent with each of the muscles whose action is in any way connected with that of the orbicularis oris, if we would thoroughly comprehend the matter. The dentist who constructs artificial teeth has in his keeping the making or marring of the whole human face. If he is not an anatomist, and at the same time has no artistic ideas, and if he does not (as carefully as the sculptor) study the face which he is endeavoring to idealize, he is unworthy a place among artistic dentists. When one sees the perverted, distorted, deformed features of some one who might have a *pleasant* expression; when children, perhaps, look upon a mother from whose hallowed image all the sweetness, love, patience and tenderness have been eliminated by the cursed work of some pretender to knowledge of which he is in utter ignorance; when we reflect upon the blasted lives of young women whose future perhaps is wrecked through their being made repulsive to one who should have been attracted; when we observe upon the streets, in society—at home and abroad—the horrible caricatures of the human face divine, that are the results of reprehensible ignorance of pretended dentists, one wonders whether, after all, our profession, as a whole, brings more of good than evil upon mankind. When we think of what it might be, and what it is, we are moved to weep at the present status of the art of dentistry, and are consoled only by the remembrance that amid all the "Cheap Johns" who are violently struggling for a bare existence, there are a few who do honor to their profession by conscientious and intelligent work, and who are rewarded—as such men always are—not only with the plaudits of a judicious world, but by the more substantial benefits of well-paid labors.

